# Accuracy of genomic selection for a sib-evaluated trait using identity-by-state and identity-by-descent relationships

**DOI:** 10.1186/s12711-014-0084-2

**Published:** 2015-02-25

**Authors:** Sergio Vela-Avitúa, Theo HE Meuwissen, Tu Luan, Jørgen Ødegård

**Affiliations:** Institute of Animal and Aquacultural Sciences, Norwegian University of Life Sciences, Ås, NO 1432 Norway; Nofima, P.O. Box 210, Ås, NO 1431 Norway; AquaGen AS, Sluppen, P.O. Box 1240, 7462 Trondheim, NO Norway

## Abstract

**Background:**

GBLUP (genomic best linear unbiased prediction) uses high-density single nucleotide polymorphism (SNP) markers to construct genomic identity-by-state (IBS) relationship matrices. However, identity-by-descent (IBD) relationships can be accurately calculated for extremely sparse markers. Here, we compare the accuracy of prediction of genome-wide breeding values (GW-BV) for a sib-evaluated trait in a typical aquaculture population, assuming either IBS or IBD genomic relationship matrices, and by varying marker density and size of the training dataset.

**Methods:**

A simulation study was performed, assuming a population with strong family structure over three subsequent generations. Traditional and genomic BLUP were used to estimate breeding values, the latter using either IBS or IBD genomic relationship matrices, with marker densities ranging from 10 to ~1200 SNPs/Morgan (M). Heritability ranged from 0.1 to 0.8, and phenotypes were recorded on 25 to 45 sibs per full-sib family (50 full-sib families). Models were compared based on their predictive ability (accuracy) with respect to true breeding values of unphenotyped (albeit genotyped) sibs in the last generation.

**Results:**

As expected, genomic prediction had greater accuracy compared to pedigree-based prediction. At the highest marker density, genomic prediction based on IBS information (IBS-GS) was slightly superior to that based on IBD information (IBD-GS), while at lower densities (≤100 SNPs/M), IBD-GS was more accurate. At the lowest densities (10 to 20 SNPs/M), IBS-GS was even outperformed by the pedigree-based model. Accuracy of IBD-GS was stable across marker densities performing well even down to 10 SNPs/M (2.5 to 6.1% reduction in accuracy compared to ~1200 SNPs/M). Loss of accuracy due to reduction in the size of training datasets was moderate and similar for both genomic prediction models. The relative superiority of (high-density) IBS-GS over IBD-GS was more pronounced for traits with a low heritability.

**Conclusions:**

Using dense markers, GBLUP based on either IBD or IBS relationship matrices proved to perform better than a pedigree-based model. However, accuracy of IBS-GS declined rapidly with decreasing marker densities, and was even outperformed by a traditional pedigree-based model at the lowest densities. In contrast, the accuracy of IBD-GS was very stable across marker densities.

## Introduction

Selective breeding programs are traditionally based on best linear unbiased prediction (BLUP) of individual breeding values that uses Henderson’s mixed model equations for an animal model [1] that use the numerator (pedigree-based) relationship matrix (**A**).

Genomic selection (GS) is a method that uses dense marker genotypes that cover the genome combined with phenotypic data to predict the breeding value of all genotyped individuals [2]. This method has proven to increase accuracy compared with traditional pedigree-based prediction of breeding values [2-4]. Several methods for GS exists, with the simplest one being the genomic BLUP (GBLUP) model, for which all marker loci are *a priori* assumed to harbor equal amounts of genetic variation [2]. An equivalent method uses dense markers to construct a genomic relationship matrix (**G**), that replaces the numerator relationship matrix in a traditional animal model [5,6]. In **G**, the relationships reflect the actual proportion of marker alleles shared by identity-by-state (IBS), as a deviation from the expected proportion of alleles shared in the population. One of the advantages of this GBLUP model is that it fits well within the existing framework of breeding value estimation.

The GS methodology is of particular relevance for traits that cannot be measured directly on selection candidates. Examples of such traits are carcass traits, sex-limited traits and disease resistance traits. The main advantage of GS for such traits lies in the fact that it allows prediction of individual breeding values for non-phenotyped (albeit genotyped) individuals, even in the absence of progeny-testing.

Compared with pedigree-based methods, GBLUP does not have any clearly defined base population, since the method potentially uses non-recorded relationships that originate from common ancestors prior to the known pedigree. Although GS in principle does not use pedigree information, it does use the available pedigree structure of the population, since the markers also capture close genetic relationships [7].

Successful implementation of GS using IBS information requires dense marker data [2] on large numbers of animals (or at least dense genotypes that can be accurately imputed), which may imply substantial genotyping costs. An alternative variant of GS uses genomic data to calculate identity-by-descent (IBD) relationships, and has led to promising results on real data [4]. This method uses marker information to trace IBD inheritance of chromosomal haplotypes within the known pedigree, using linkage analysis through the LDMIP method [8], a methodology originally conceived to phase genotypes. The LDMIP method uses an iterative peeling step for each genotyped locus, which accounts for family information, and adjusts transmission probabilities for the information from neighboring loci using a forward-backward algorithm. The accuracy of this method depends on the availability of extensive family data and family structure [8]. Due to the limited number of recombinations that occurs from one generation to the next, this approach does not require dense markers, which is why it has been used to perform genetic analyses with sparse markers [9]. The IBD relationship matrix is expected to be robust to marker density but the effect of marker density on accuracy of estimated breeding values using the IBD relationship matrix has not been quantified. Reducing marker density may also reduce costs, either through genotyping with less costly low-density SNP marker panels, or through new technologies such as genotyping-by-sequencing.

The genomic IBD approach for breeding value estimation is based on some of the same fundamental assumptions as the traditional pedigree-based analysis: (1) a clearly defined base population, in which all base animals are considered unrelated; (2) the base population provides a reference point for the genetic mean and variance; (3) all IBD relationships exist through the pedigree; and (4) the genetic covariance between animals is assumed proportional to their IBD relationship.

The iterative nature of the LDMIP method uses the actual inheritance of genome-wide markers available for (a genotyped fraction of) the population, combined with prior knowledge about the pedigree. Furthermore, IBD probabilities of all animals in the pedigree are estimated, and there is thus no need to blend genomic and pedigree-based matrices. The classical pedigree-based analysis is actually a special case of the genomic IBD approach, i.e., assuming the fraction of genotyped individuals to be zero and thus estimating IBD relationships based on prior information only. Hence, for real data, any inclusion of genome-wide (sparse to dense) marker information on (a fraction of) the population is expected to improve the estimated IBD relationship matrix compared to the use of pedigree information alone.

The main objective of this work was to compare accuracy of BLUP breeding values calculated using three methods to estimate additive genetic relationships: (1) pedigree-based relationships (**A** matrix), (2) IBS sharing of marker alleles (**G**_**IBS**_ matrix), and (3) IBD sharing of marker alleles (**G**_**IBD**_ matrix). Methods were compared for a typical aquaculture breeding scheme using stochastic simulation.

## Methods

### Simulations

**Figure 1:**
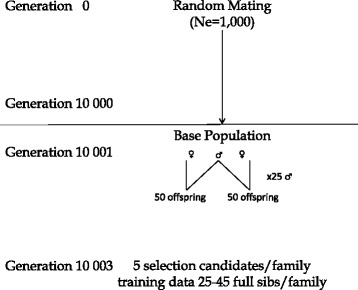
**Scheme of the simulated genetic model.**

Simulations were performed with the QMSim simulation software [10], using the simulation scheme of Figure 1. A base population was generated by simulating a population over 10 000 generations with a constant effective population size of 1000 individuals, using random mating. In generation 10 000, a base population was constructed, consisting of 50 sires and 25 dams (each sire mated with two dams), all randomly selected. Each female produced 50 offspring (100 offspring per male). The same structure was repeated over three generations. Pedigree, phenotypes, QTL (quantitative trait locus) effects and genotypes were recorded for the last three generations only.

The genome consisted of 20 chromosomes 1 Morgan (M) long. For each chromosome, 20 001 markers and 20 000 QTL (one QTL between each pair of neighboring markers) were simulated. Mutation rate was set to μ = 5×10^−6^ for markers and to μ = 1×10^−6^ for QTL. In generation 10 000, all segregating QTL were used in the analysis (~1300 QTL/chromosome); while only markers with two alleles (SNP-like) and a minor allele frequency greater than 0.05 were used in the analysis (~1200 markers/chromosome). All genetic variance was assumed due to additive QTL effects, which were randomly sampled from a gamma distribution with shape parameter 0.4 and scaled so that the genetic mean was equal to 0 and genetic variance was equal to 0.3 in the base population in each replicate. Phenotypes were generated by adding random residuals to the genetic effects, which were sampled from independent normal distributions $\sim N\left(0,\mathbf{I}{\sigma}_e^2\right)$. Heritabilities in the base population of 0.1, 0.3 and 0.8 were simulated by scaling the residual variance accordingly (σ_e_^2^ = 2.7, 0.7, and 7.5*10^−2^, respectively). Hence, the same individuals, genotypes, genetic effects and unscaled residuals were used in parallel replicates for the different heritabilities. A total of 50 replicates were generated for each scenario.

### Sampling of markers for genetic analyses

Sub-samples of markers containing 10, 20, 50, 100 and 300 markers per chromosome were generated in the following way: chromosomes were divided into equal segments (in M) according to the desired number of markers in the sub-sample, and a single marker was randomly sampled from each segment. Hence, all segments of the genome were covered, but the sampled markers were not exactly equidistant.

### Estimation of breeding values

Breeding values were predicted by solving Henderson’s mixed model equations, using the R environment [11]. In addition to traditional pedigree-based breeding values (EBV), genomic breeding values (GW-EBV) were predicted using either IBS or IBD genomic relationship matrices. For all three methods, the general statistical model was:

$$ \mathbf{y}=1\mu +\mathbf{Z}\mathbf{a}+\mathbf{e}, $$

where **y** is a vector of phenotypes; *μ* is the overall mean; **Z** is a design matrix that links the animals to the records; **a** is a vector of breeding values of the animals and **e** is a vector of random residuals. It was assumed that **a** *~ N* (**0**, **G***σ*^2^_*a*_) where *σ*^*2*^_*a*_ is the additive genetic variance and **G** the relationship matrix constructed either as **G**_**IBS**_ (IBS relationship matrix), **G**_**IBD**_ (IBD relationship matrix), or **A** (numerator relationship matrix for traditional pedigree-based analysis).

### Calculation of genomic relationship matrices

#### IBS relationship matrix

For IBS genomic selection (IBS-GS), the IBS relationship matrix was calculated following VanRaden [6]:

$$ \mathbf{G}=\mathbf{W}\mathbf{W}\hbox{'}/N, $$

where *N* is the number of markers and **W** is a matrix with standardized genotypes in the form of:

$$ {w}_{ij}=\frac{x_{ij}-2{\mathrm{p}}_j}{\sqrt{2{\mathrm{p}}_j\left(1-{p}_j\right)}}, $$

where *x*_*ij*_ is the genotype of individual *i* at marker *j,* denoted as 0, 1 or 2 for the homozygote, heterozygote and the other homozygote, respectively, and *p*_*j*_ is the allele frequency at marker *j* in the base population*.* A small value of 10^−4^ was added to the diagonal elements of **G** in order to secure that the matrix was positive definite.

#### IBD relationship matrix

For IBD genomic selection (IBD-GS), the IBD relationship matrix was constructed using the approach of Fernando and Grossman [12]. Paternal (and maternal) conditional genotype inheritance probabilities were calculated as proposed by Meuwissen *et al.* [13], in which genotype probabilities and segregation indicator probabilities are estimated using the multilocus iterative peeling methodology (LDMIP) of Meuwissen and Goddard [8]. Here, IBD relationship matrices were calculated for each marker, and the genome-wide **G**_**IBD**_ matrix was obtained by averaging these matrices over all markers. As in the IBS relationship matrix, a small value was added to all diagonal elements to secure that the matrix was positive definite.

### Model comparison

Genomic selection is particularly valuable when predicting breeding values of genotyped individuals without own phenotype or offspring information. Hence, the methods were compared on how accurately they predicted the true breeding values of subsets of animals without own phenotypes. These subsets were created by randomly masking the phenotypes of five individuals from each family in the last generation (validation animals). Furthermore, the effect of the number of phenotyped and genotyped sibs was investigated by masking additional random sibs from each family, i.e., the genetic evaluation of the validation animals was based on phenotypes and genotypes of training samples consisting of 50 to 90% (in 10% steps) of the remaining family members (i.e., 25 to 45 sibs). Breeding values were then estimated using the different models and marker densities. The predictive ability of each model was estimated as the Pearson’s correlation coefficient (*r)* between predicted breeding values and true breeding values of the validation individuals with masked phenotypes (5 sibs/family and 50 families).

## Results

**Figure 2:**
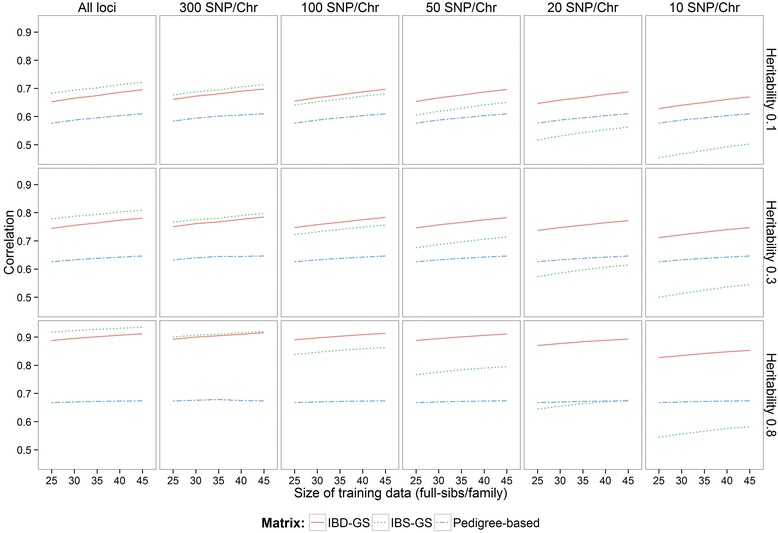
**Correlations between true and predicted breeding values for all marker densities (~1200 SNP/M, 300 SNP/M, 100 SNP/M, 50 SNP/M, 20 SNP/M and 10 SNP/M) and size of training data (25–45 full sibs/family).**

Results averaged over replicates for the pedigree-based, IBD-GS and IBS-GS models are in Figure 2. At the highest marker density, both IBD-GS and IBS-GS were clearly more accurate than pedigree-based EBV, and in some cases, accuracy was greater by more than 20%. Furthermore, the relative advantage of GS methods increased with increasing heritability; at the highest density, IBS-GS was 11% more accurate than the pedigree-based method when heritability was 0.1, while with a heritability of 0.8, the corresponding difference was as large as 26%. Similar results were obtained for the IBD-GS model, which had 9% and 24% greater accuracies than the pedigree-based method for heritabilities of 0.1 and 0.8, respectively.

The IBS-GS method was more accurate than IBD-GS at the two highest marker densities (2.4 to 3.1%) and was outperformed by IBD-GS only at densities lower than 300 SNPs/M. At the highest marker density, the advantage of IBS-GS relative to IBD-GS was greater than at the lowest heritability (~4.3%). However, most striking were the large differences between IBD-GS and IBS-GS in their sensitivity to marker density: For IBD-GS, there was hardly any reduction in accuracy when marker density was decreased from ~1200 to 50 SNPs/M, and the method performed well for as few as 10 SNPs/M (2.5 to 6.1% reduction in accuracy compared to ~1200 SNPs/M). In contrast, the accuracy of IBS-GS decreased with any reduction in marker density, and at the lowest marker densities (10 to 20 SNPs/M), IBS-GS was even clearly outperformed by pedigree-based BLUP. Losses in accuracy due to reductions in marker densities were more pronounced for traits with higher heritabilities. Thus, losses in accuracy associated with incorrect or imprecise relationships were greatest for highly heritable sib-evaluated traits.

The IBS-GS and IBD-GS methods were equally affected by size of training datasets. When decreasing the number of training sibs per family from 45 to 25, both models had a similar decrease in accuracy, ranging from 2.3 to 4.2% for IBD-GS and from 1.7 to 4.9% for IBS-GS, while the pedigree-based model was more robust, with reductions in accuracy ranging from only 0.5 to 3%. As expected, reductions in accuracy associated with reductions in training data size were more pronounced for traits with low heritabilities.

## Discussion

At the highest marker density, IBS-GS performed slightly better than IBD-GS and both methods performed considerably better than the traditional pedigree-based evaluations. This agrees with results obtained by Luan *et al.* [4] with dairy cattle data and using ~35 000 SNPs. That study found that when including at least four generations of pedigree to calculate the **G**_**IBD**_ matrix, precision of EBV of bulls from an IBD-GS model were similar to those from an IBS-GS model. In the present work, only three generations were included to calculate the **G**_**IBD**_ matrix, which resulted in a slight advantage for IBS-GS at the highest marker density. We attribute this slightly better predictive ability of IBS-GS to the inclusion of LD information in the prediction of breeding values, although this may also be a result of the rather simplified assumptions made in the stochastic simulation. Real data is likely more complex, for example LD patterns may be affected by patterns in recombination rates, as reported in Atlantic salmon (*Salmo salar*) [14], where the recombination rate in males is significantly lower than in females, as well as by admixture events, since aquaculture populations are typically based on multiple (wild) founder populations e.g. [15].

According to Habier *et al.* [16], three types of quantitative genetic information contribute to the accuracy of genomic selection models: (1) pedigree information, (2) co-segregation and (3) population-wide LD. The first source of information is likely covered by all three models. The pedigree-based and IBD-GS models both use the pedigree directly, while IBS-GS uses marker genotypes, which are inherited from parents. However, marker-based relationships may be imprecise measures of ancestry when they are based on the lowest marker densities, which may explain the poor performance of IBS-GS in these scenarios.

Co-segregation is a deviation from independent segregation on the same gamete of linked loci and GS models can capitalize on these (small) deviations from expected IBD relationships. These deviations can be captured by linkage analysis when using the IBD-GS model, but are ignored in the pedigree-based model. Co-segregation information may also be captured by IBS-GS, since IBD relationships necessarily also imply a relationship at the marker level (but not necessarily vice versa). Still, an accurate representation of the relationships requires dense marker data, which contributes to the sensitivity of IBS-GS to marker density. Furthermore, since co-segregation can be defined as within-family deviations from expected relationships, the relative importance of this information is expected to increase with family size and heritability (i.e., when the Mendelian deviations from the family mean are expected to have substantial effect on the phenotypes). This agrees with the results of this study, which shows increased potential (accuracy) of IBD-GS and IBS-GS models (at high marker density) with increasing heritability and size of the training dataset (family size).

LD information is only captured by IBS-GS, provided that the marker density is sufficiently high, which may explain the slight superiority of IBS-GS over IBD-GS at the highest marker density. LD information can be viewed as a general association between specific marker genotypes and phenotypes across the population. Since these are population-wide effects, their estimation may be less sensitive to heritability, which may explain the relative higher superiority of IBS-GS over IBD-GS for traits with low heritability (at the higher marker density).

At high marker densities, construction of the **G**_**IBD**_ is computationally very demanding, with computer time increasing linearly with number of markers and number of individuals. However, the **G**_**IBD**_ matrix is less dense than the corresponding **G**_**IBS**_ matrix, since all relationships between animals that are not related within the known pedigree are set to 0. This reduces dependencies and thus simplifies computation of the inverse of the relationship matrix. In addition, the LDMIP method is an optimal way of including relationship information, combining ungenotyped and (dense to sparsely) genotyped animals into a common relationship matrix.

## Conclusions

Both IBS-GS and IBD-GS models for the prediction of the breeding value of a sib-evaluated trait proved to perform better than the pedigree-based model in a population with strong family relationships. IBS-GS was slightly more accurate than IBD-GS at the highest marker densities, but also considerably more sensitive to a reduction in density. In contrast, accuracy of IBD-GS was very stable across marker densities, with minimal losses in accuracy even at extremely low densities, and always had considerably greater accuracy than the pedigree-based analysis (even at 10 SNPs/M). Hence, the IBS-GS model has the highest accuracy, but requires dense marker data, which may be costly to obtain. Sparse genotyping combined with imputation may be a viable alternative, but requires access to high-density marker data and genetic maps, which may not be available in most aquaculture species in the near future. In such situations the IBD-GS model may be a viable alternative.
